# Characterization of the LPS and 3OHFA Contents in the Lipoprotein Fractions and Lipoprotein Particles of Healthy Men

**DOI:** 10.3390/biom12010047

**Published:** 2021-12-29

**Authors:** Pere Rehues, Marina Rodríguez, Judith Álvarez, Marta Jiménez, Alba Melià, Mar Sempere, Clara Balsells, Gemma Castillejo, Montse Guardiola, Antoni Castro, Josep Ribalta

**Affiliations:** 1 Unitat de Recerca en Lípids i Arteriosclerosi, Departament de Medicina i Cirurgia, Universitat Rovira i Virgili Reus, 43003 Tarragona, Spain; pere.rehues@urv.cat (P.R.); marina.rodriguez@alumni.urv.cat (M.R.); alvarez.judith@hotmail.com (J.Á.); jimenezescrig.marta@gmail.com (M.J.); albamelseg@gmail.com (A.M.); mar.semca@gmail.com (M.S.); clarabalsells@gmail.com (C.B.); josep.ribalta@urv.cat (J.R.); 2 Institut d’Investigació Sanitària Pere Virgili, 43204 Reus, Spain; gemma.castillejo@urv.cat (G.C.); antoni.castro@urv.cat (A.C.); 3 Centro de Investigación Biomédica en Red de Diabetes y Enfermedades Metabólicas Asociadas, 28029 Madrid, Spain; 4 Unitat de Gastroenterologia Pediàtrica, Hospital Universitari Sant Joan de Reus, 43204 Reus, Spain; 5 Unitat de Malalties Autoinmunes, Medicina Interna, Hospital Universitari Sant Joan de Reus, Universitat Rovira i Virgili, 43204 Reus, Spain

**Keywords:** lipopolysaccharide, lipoprotein, 3-hydroxy fatty acids, atherosclerosis

## Abstract

Atherosclerosis is a chronic inflammatory disease that is caused by the accumulation of LDL particles in the intima, causing the activation of immune cells and triggering an inflammatory response. LPS is a potent activator of the innate immune response and it can be transported by lipoproteins. Since humans are much more sensitive to LPS than other mammals, and very low amounts of LPS can elicit an immune response, the aim of this study is to characterize the distribution of LPS and its immunogenic portion (3OHFAs) among lipoprotein types of healthy men. We separated lipoprotein fractions by ultracentrifugation and the amount of each 3OHFA was measured by MS in each lipoprotein fraction to calculate LPS concentration. Lipoprotein particle concentration was measured by NMR. LDL and HDL fractions transported the highest concentration of LPS (35.7% and 31.5%, respectively), but VLDL particles carried more LPS molecules per particle (0.55 molecules/particle) than LDL or HDL (*p* < 0.01). The distribution of LPS and all 3OHFAs among lipoprotein fractions showed high interindividual variability, suggesting that they may be studied as a potential biomarker. This may help understand the role of LPS in atherosclerosis in those cases where the disease cannot be explained by traditional risk factors.

## 1. Introduction

Atherosclerosis is a chronic inflammatory condition resulting from arterial haemodynamic changes and complex interactions between a variety of cell types, lipids and soluble mediators [1]. To date, many risk factors for atherosclerosis have been identified, such as the relative amount of each lipoprotein type in plasma, familial history, systemic inflammation, a high-fat diet, tobacco and infections [2]. However, the etiopathology of atherosclerosis is complex, and the potential roles of several other factors have not yet been elucidated. Moreover, between 30% and 40% of complications derived from atherosclerosis occur in the absence of these risk factors, meaning that other still unknown causes may be involved. Identifying these other risk factors and their role could help understand the development and outcome of atherosclerosis in people where the traditional risk factors cannot explain the disease; additionally, these patients are often unresponsive to common treatments. This paper focuses on one of these new risk factors, lipoprotein-bound circulating lipopolysaccharides (LPS) [3,4,5].

LPS, also known as endotoxin, is the major glucolipid component of the outer membrane of Gram-negative bacteria [6]. LPS from the gut microbiota can reach the bloodstream either by being filtered through the intestinal wall as a consequence of increased gut permeability and impaired tight junctions (paracellular transport) or by joining chylomicrons (transcellular transport) [7]. Once in the bloodstream, LPS can be transferred to other lipoprotein types [7,8].

LPS is an amphipathic molecule with a variable hydrophilic motif consisting of a polysaccharide chain (O antigen) and a conserved hydrophobic domain (lipid A). Lipid A is composed of a disaccharide esterified with four 3-hydroxy fatty acids (3OHFAs). Lipid A is very conserved among bacterial species and is the bioactive portion of the LPS molecule that is responsible for the activation of proinflammatory pathways and involved in the innate immune response [6].

Low levels of LPS can trigger the production of high levels of proinflammatory endogenous mediators; on the other hand, chronic and systemic exposure to slightly high LPS concentrations can lead to subclinical chronic inflammation [9], a process that is related to a higher metabolic risk. Patients with a high cardiovascular risk, such as those with obesity or diabetes, have increased plasma LPS levels compared to healthy controls [9,10,11], thus suggesting a possible association between LPS and metabolic disorders [12]. Additionally, baseline LPS levels are a risk factor for the incidence of atherosclerosis [13].

In circulation, LPS is mostly bound to lipoproteins (between 80% and 97% of total LPS) [14,15]. This binding may be mediated by LPS-binding protein (LBP), which has a great affinity for LPS, can bind to lipoproteins and has been reported to enhance the association of LPS with ApoB-containing lipoproteins [16,17]. However, little is known about the distribution of LPS among the different lipoprotein types, and there is some controversy among previous reports addressing this issue [14,16,18,19].

Given the lack of consensus, we believe that more in-depth studies on the distribution and transport of LPS by lipoproteins are needed. In the existing literature, different methods have been used for lipoprotein separation and LPS quantification. In our study, we measured LPS by quantifying 3OHFAs by mass spectrometry, which is reliable and precise and can provide information about the fatty acid chains that are bound to the glucosamine backbone of lipid A [20], the immunogenic part of LPS. The objective of this work was to quantify the LPS bound to each lipoprotein type in a group of apparently healthy people.

## 2. Material and Methods

### 2.1. Volunteers

Twenty-five apparently healthy young men, older than 18 years of age, were recruited, all of whom presented a normal lipid profile and had no chronic diseases. The experimental protocol was approved by the hospital’s ethical committee, and all participants signed an informed consent form.

### 2.2. Biochemical Analyses

Fasting venous blood samples were collected in EDTA tubes and centrifuged immediately for 15 min at 1500× *g* and 4 °C to obtain plasma. The samples were then divided into aliquots and stored at −80 °C until analysis.

Standard laboratory methods were used to quantify the total cholesterol, triglycerides and HDL cholesterol contents. LDL cholesterol values were calculated using the Friedewald formula [21]. Apolipoproteins were quantified using an immunoturbidimetric assay with specific antibodies against apolipoprotein A1 (Apo A1), apolipoprotein B100 (Apo B100) and apolipoprotein CIII (Apo CIII). These analyses were adapted for the Cobas-Mira-Plus autoanalyzer (Roche Diagnosis, Barcelona, Spain).

### 2.3. Lipoprotein Separation

Lipoprotein fractions were separated by sequential ultracentrifugation after progressively increasing the solvent density using an Optima XPN-100 ultracentrifuge (Beckman Coulter, San Jose, CA, USA) and a Kontron 45.6 fixed-angle rotor at 37,000 rpm and 4 °C for different time intervals: 16 h for chylomicrons and VLDL, 20 h for IDL, 20 h for LDL and 40 h for HDL. To separate these fractions, different solutions were used to set the densities of the samples to 1006, 1019, 1063 and 1210 g/mL.

### 2.4. LPS Determination

The LPS concentration was determined by quantifying the amount of 3OHFAs by gas chromatography coupled to triple quadrupole mass spectrometer (GC-QqQ/MS). 3OHFAs are anchored to a phosphorylated glucosamine disaccharide and are chemical markers of endotoxin [22]. In this work, 3OHFAs with chain lengths of 8–18 carbons were detected in samples using a method based on the alkaline hydrolysis of LPS, trimethylsilyl derivatization of the obtained 3OHFAs and analysis by GC-QqQ/MS. Analysis was performed on a 7890A Series gas chromatograph coupled to a 7000 GCQqQ (Agilent Technologies, Santa Clara, CA, USA). Chromatographic column was a J&W Scientific HP5-MS (30 m × 0.25 mm i.d., 0.25 μm film) (Agilent Technologies).

Since 3OHFA products from mitochondrial fatty acid β-oxidation can represent a limitation that interferes with the analysis [23], total and free 3OHFAs were analysed, and the 3OHFA concentration from LPS was obtained as the difference between the two concentrations. Finally, the total LPS content was calculated as the sum of the number of nanomoles of the individual 3OHFAs divided by 4 to account for the four 3OHFA molecules assumed to be present per molecule of LPS [24].

### 2.5. Liposcale^®^ Test: Nuclear Magnetic Resonance (NMR) Lipoprotein Profile

Total plasma lipids and the lipoprotein subclass distributions were analysed by NMR spectroscopy by Biosfer Teslab (Reus, Spain). The Liposcale^®^ test is a novel advanced lipoprotein test based on 2D diffusion-ordered 1H-NMR spectroscopy using diffusion coefficients [25]. This technique provides the particle size and number of the main types of lipoproteins (VLDL, LDL, and HDL) and divides each type into large, medium and small sizes according to the increases in molecular weight. Moreover, 1H-NMR approaches based on regression methods allow for the determination of the cholesterol and triglyceride concentrations of each lipoprotein subclass [26]. 1H-NMR was carried out on EDTA plasma which was stored and thawed prior to analysis.

### 2.6. Statistical Analysis

Statistical analysis was carried out using SPSS Statistical Software version 27 (SPSS Inc., Chicago, IL, USA) and GraphPad Prism (version 5). The normal distribution of the variables was assessed using the Shapiro–Wilk test, differences between variables were determined using the Kruskal–Wallis test with Dunn’s post-test. Statistical significance was set at *p* < 0.05.

## 3. Results

We studied 25 young healthy males. The anthropometric and conventional lipid profile data showed that all parameters were within the normal range (Table 1).

### 3.1. LPS Was Found in All Lipoprotein Particles with High Interindividual Variability

**Figure 1:**
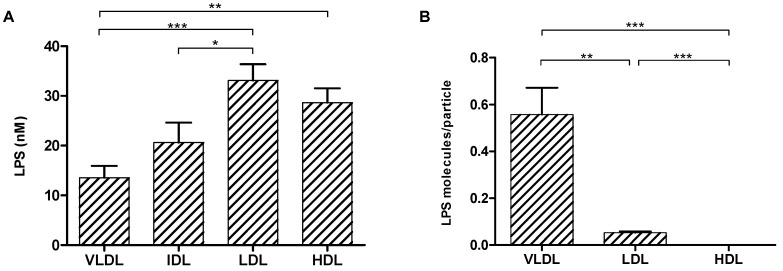
(**A**) Mean concentration (nM) of LPS in VLDL, IDL, LDL and HDL fractions in the study group. (**B**) Mean LPS/particle ratio in VLDL, LDL and HDL. * *p* < 0.05, ** *p* < 0.01 and *** *p* < 0.001.

LPS was quantified in all plasma and lipoprotein fractions from a group of 25 healthy young men. Figure 1 shows that all lipoproteins transport LPS. The average contents of LPS in the lipoprotein particles showed the highest values in the LDL (33.12 nM, 35.7%) and HDL (28.65 nM, 31.5%) fractions, followed by IDL (20.60 nM, 18.9%) and VLDL (13.56 nM, 13.9%) (*p* < 0.0001) (Figure 1A). The total concentration of LPS was variable between subjects, and analysis of the distribution of LPS between lipoproteins in each participant also showed very high interindividual variability (Supplementary Materials Figure S1).

### 3.2. LPS Molecules per Lipoprotein Particle

The number of VLDL, LDL and HDL particles was measured in each individual using the Liposcale test (Supplementary Materials Figure S2), and the number of LPS molecules per particle ratio was computed. While VLDL was the lipoprotein that carried the lowest concentration of LPS, VLDL carried the highest mean number of LPS molecules per particle (0.55 molecules/particle), followed by LDL (0.05 molecules/particle) and HDL (less than 0.01 molecules/particle). Significant differences between the means were observed (*p* < 0.0001) (Figure 1B).

### 3.3. OHFAs

As the bioactive part of the LPS molecule, 6 types of 3OHFAs (with different carbon chain lengths) belonging to the lipid A portion of the LPS molecule were quantified, and the distribution of all 6 types of fatty acids among all lipoprotein types was analysed.

**Figure 2:**
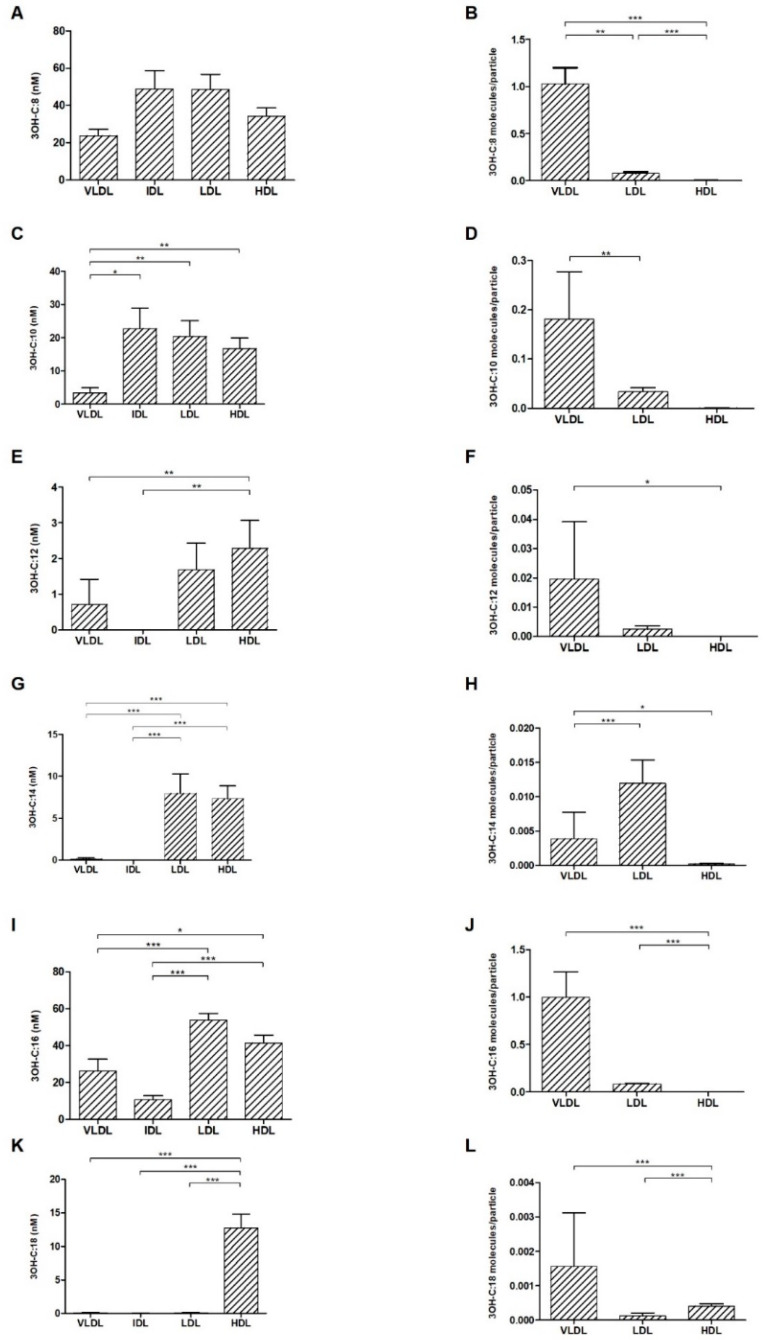
Mean concentration (nM) of each LPS-derived 3OHFA, namely 3OH-C:8 (**A**), 3OH-C:10 (**C**), 3OH-C:12 (**E**), 3OH-C:14 (**G**), 3OH-C:16 (**I**) and 3OH-C:18 (**K**) in VLDL, IDL, LDL and HDL fractions. Mean 3OHFA/particle ratio of 3OH-C:8 (**B**), 3OH-C:10 (**D**), 3OH-C:12 (**F**), 3OH-C:14 (**H**), 3OH-C:16 (**J**) and 3OH-C:18 (**L**) in VLDL, LDL and HDL fractions. * *p* < 0.05, ** *p* < 0.01 and *** *p* < 0.001.

3OH-C:8, 3OH-C:10 and 3OH-C:16 were the fatty acids with the highest concentrations, and these fatty acids were present in all lipoprotein fractions. On the other hand, 3OH-C:12 and 3OH-C:14 were present in only HDL and LDL (in one subject, also in VLDL) and 3OH-C:18 was detected in only HDL (except for three subjects that had a low amount of 3OH-C:18 in VLDL, IDL or LDL) (Figure 2A,C,E,G,I,K). High variability between 3OHFA concentrations between study subjects was also observed (Supplementary Figures S3–S8).

The number of 3OHFA molecules per lipoprotein particle was also calculated. Similar to the results obtained with LPS, VLDL was the lipoprotein particle that transported more 3OHFA molecules, particularly 3OH-C:8, 3OH-C:10 and 3OH-C:16 (in contrast, LDL had a higher load of 3OH-C:14 molecules per particle) (Figure 2B,D,F,H,J,L).

In order to explore those factors that might be affecting LPS and 3OHFA lipoprotein content, we studied their correlation with BMI and age.

BMI does not correlate significantly with any of the lipoprotein-LPS measurements but it shows an inverse and statistically significant correlation with LDL 3OH C:8 (r = −0.425; 0.038). Conversely, VLDL 3OH C:16 (r = 0.594; *p* = 0.003) and LDL 3OH C:14 (r = 0.405; *p* = 0.049) show a significant positive correlation with age.

## 4. Discussion

Atherosclerosis is an inflammatory process of the artery that leads to cholesterol accumulation and cardiovascular disease. We hypothesized that LPS may help to explain accelerated atherosclerosis, and the reason for this is threefold: (1) LPS binds to lipoproteins for its elimination; therefore, lipoproteins located in the subendothelial space may contain LPS; (2) unlike most mammals, humans have developed a very high immunomodulatory response to LPS linked to the acyl chains of lipid A [27]; (3) LPS has been found in the carotid plaques of patients with atherosclerosis [28]. Therefore, an exacerbated immune response leading to foam cell formation may be triggered by LPS even at similar concentrations of circulating cholesterol.

LPS from the gut microbiota enters the bloodstream via intestinal absorption and binds to chylomicrons, and once in the bloodstream, LPS can be transferred to other lipoproteins [7,8]. Given that most of the LPS in circulation is transported by lipoproteins [14], we aimed to characterize LPS distribution in plasma and in each of the lipoprotein types (VLDL, IDL, LDL and HDL) of healthy subjects as a specific biomarker for future epidemiological studies exploring atherosclerosis risk. Moreover, we also characterized the individual composition of each lipoprotein with respect to the most immunomodulatory portion of the LPS molecule, the lipid A acyl chains.

Our results showed that in healthy subjects, there was high interindividual variability in the distribution of LPS among lipoprotein fractions, which was also observed in previous reports [13]. In our population, LPS was mainly transported by LDL (35.7%) and HDL (31.5%). Assessing the distribution of LPS in lipoprotein fractions is a controversial issue. A study by Vergès et al. reported that in healthy people, LDL had higher (55%) LPS contents than VLDL (22%) or HDL (19%) [19], and van Lenten et al. suggested that LPS is transported, in general, by LDL particles [18]. On the other hand, Levels et al. reported that in 10 healthy people, LPS was mainly bound to HDL (60%), and lower amounts were found in the LDL and VLDL fractions (25% and 12%, respectively) [14]. Finally, other studies have stated that LPS is mainly found in VLDL and LDL [16,29]. In summary, most studies seem to agree that LDL or HDL are the main lipoproteins involved in LPS transport. These studies have used different LPS determination techniques, such as Limulus amebocyte lysate (LAL) or the labelling of LPS molecules in in vitro studies. Although the relative LPS contents in the lipoprotein fractions are comparable to our results, there are some discrepancies in the absolute concentration values, which may be explained by methodological differences. Other studies that have used methodologies similar to ours [22] have reported values that are comparable to those obtained in the present article.

**Figure 3:**
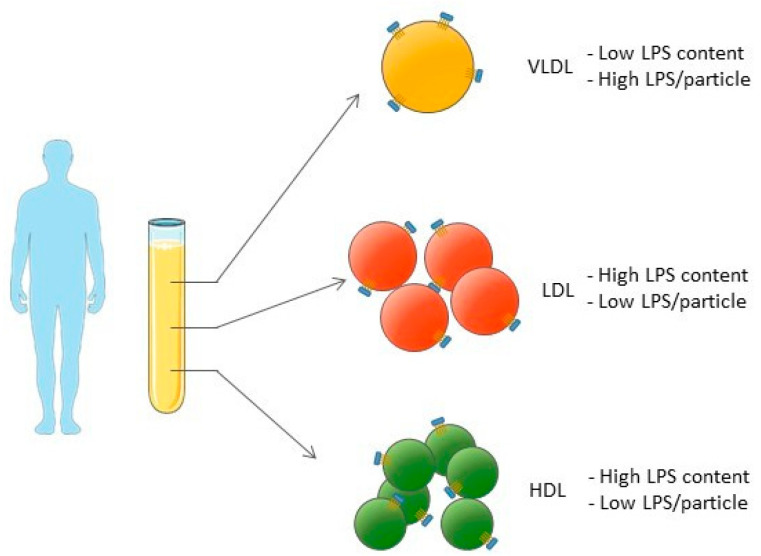
Summarized distribution of LPS among lipoproteins.

As we also measured the lipoprotein particle concentration by NMR, we were able to determine the LPS concentration per lipoprotein particle. While the whole VLDL fraction contains a very low concentration of LPS compared to the LDL or HDL fractions, each VLDL particle carries 10 and 50× more LPS molecules than one LDL or HDL particle, respectively (summarized in Figure 3). This suggests that VLDL particles that pass through the artery wall would also transport more LPS molecules into the intima, increasing the proinflammatory stimuli. This potential proatherogenic capacity of VLDL is very much in line with the observation of Balling et al., who reported an important contribution of VLDL to atherosclerotic disease. According to the authors, the main reasons for the proatherogenic role of VLDL are (1) its large size, which causes VLDL remnants to become easily trapped within the intima, and (2) the direct uptake of VLDL remnants by macrophages without the need for oxidation [30]. Our results are in line with those published in the aforementioned article and suggest an additional LPS-related proatherogenic role of VLDL particles.

As mentioned above, humans are much more sensitive to LPS than other mammals, and very low amounts of LPS can cause inflammation and elicit an immune response [27]. As this immune response is mostly due to acyl chains, in our study, we also measured the distribution acyl chains in each lipoprotein. The mechanism by which the immune cascade is activated is as follows. In circulation, LPS can form a complex with LPS-binding protein (LBP). LBP can transfer LPS to CD14 (either as a soluble molecule or as a receptor in the plasma membrane) for recognition by the Toll-like receptor (TLR) 4-MD2 complex in the membrane of monocytes, macrophages and other immune cells [31]. Activation of TLR4 signaling leads to the activation of NFkB and the transcription of proinflammatory cytokines [32], which are responsible for the acute phase response to bacterial infections. LPS interacts with the TLR4–MD2 complex via lipid A. Its four 3OHFAs linked to glucosamine can be of different lengths and also have secondary acyl chains. Depending on the number of acyl chains, lipid A can be more or less immunogenic [33], and even the length of the acyl chain is suggested to interfere with LAL activity [34].

In the present article, analysis of the distribution of 3OHFAs among the lipoprotein fractions revealed that some 3OHFAs are not equally distributed among the lipoprotein fractions. This could be related to the different binding affinities of the 3OHFA types for each lipoprotein class, but more research is needed to clarify this point.

Although the study group was not selected to explore factors modulating the LPS content of individual lipoproteins, we observed a positive association between age and some lipoprotein 3OHFAs. However, BMI showed an inverse correlation with some LPS and 3OHFA parameters. As lipids and intestinal permeability increase with age such correlation is somehow expected, but the inverse correlation with BMI has to be taken with caution and deserves to be confirmed in a much larger and heterogeneous population.

This study has some limitations. The study was designed to explore whether LPS and 3OHFA could be quantified in each lipoprotein, thus we studied a group of young healthy men with presumably low concentrations of LPS. For this purpose, the study group is small but homogeneous and it was not intended to explore associations with atherosclerosis or other metabolic conditions. Further studies in patients with diagnosed atherosclerosis are necessary to evaluate the utility of LPS as a biomarker for atherosclerosis. Some aspects may affect the LPS concentration and/ or distribution among lipoproteins such as diet, smoking or the presence of asymptomatic infections, and we only had record of the smoking habits. The use of complex and expensive equipment such as GC-QqQ/MS for LPS determination may also be a limitation for its use in routine practice. In conclusion, we report the concentration of LPS and the immunogenic acyl chains 3OHFAs in each lipoprotein fraction of healthy subjects. Our results indicate that while LDL and HDL are the lipoprotein fractions that contain more LPS, the amount of LPS per particle was much higher in VLDL particles than in LDL or HDL particles, suggesting a potential atherogenic mechanism of VLDL associated with LPS that should be evaluated in other studies in patients with atherosclerosis. We observed high interindividual variability in the distribution of both LPS and 3OHFAs among lipoprotein fractions. This suggests that the LPS and 3OHFA lipoprotein content may be an informative biomarker for future population studies in diseased groups. These findings could help set a basis for a better understanding of the role of LPS in atherosclerosis and, importantly, in those cases where known risk factors are not satisfactory predictors of this pathology.

## Figures and Tables

**Table 1:** Characteristics of the study population.

Age, years	25.7 ± 5.6
Body Mass Index, kg/m^2^	23.3 ± 2.2
Smokers, n	6/25
Cholesterol, mg/dL	169.8 ± 23.1
LDLc, mg/dL	91.7 ± 22.1
HDLc, mg/dL	57.9 (52.3–68.5)
Triglycerides, mg/dL	80.5 (65.0–102.2)
ApoA1, mg/dL	140.0 ± 18.5
ApoB100, mg/dL	80.0 ± 17.1
ApoC-III, mg/dL	7.8 ± 3.3

Values are means ± SD for variables with a normal distribution or medians (IQR) for variables with no normal distribution.

## Data Availability

The data presented in this study are available on request from the corresponding author.

## Supplementary Materials

The following supporting information can be downloaded at: https://www.mdpi.com/article/10.3390/biom12010047/s1, Figure S1: LPS concentrations; Figure S2: Mean lipoprotein concentrations; Figure S3: 3OH-C:8 fatty acid concentrations; Figure S4: 3OH-C:10 fatty acid concentrations; Figure S5: 3OH-C:12 fatty acid concentrations; Figure S6: 3OH-C:14 fatty acid concentrations; Figure S7: 3OH-C:16 fatty acid concentrations; Figure S8: 3OH-C:18 fatty acid concentrations.
