# Supplementary material for: Characterization of the LPS and 3OHFA Contents in the Lipoprotein Fractions and Lipoprotein Particles of Healthy Men
Source: Biomolecules. 2021 Dec 29;12(1):47. doi: 10.3390/biom12010047 (PMC8773495; doi:10.3390/biom12010047)
Supplement: Supplementary file 1 [file biomolecules-12-00047-s001.zip › biomolecules-1517017-supplementary.pdf]

Supplemental figure S1

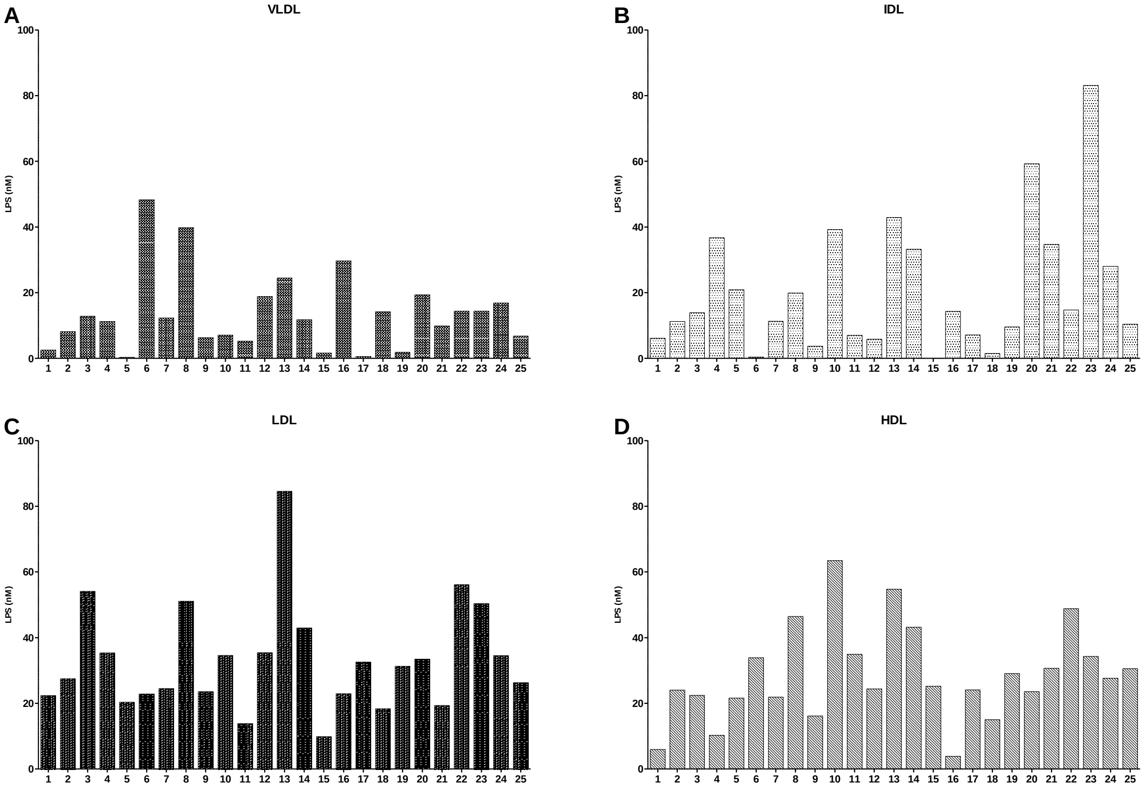

Supplemental figure S1: Individual LPS concentrations (nM) in VLDL (A), IDL (B), LDL (C) and HDL (D) fractions of all study subjects.

Supplemental figure S2

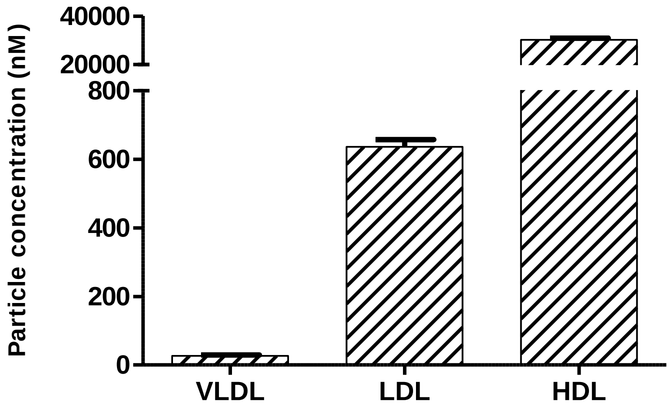

Supplemental figure S2: Mean concentration (nM) of VLDL, LDL and HDL particles in the study group.

Supplemental figure S3

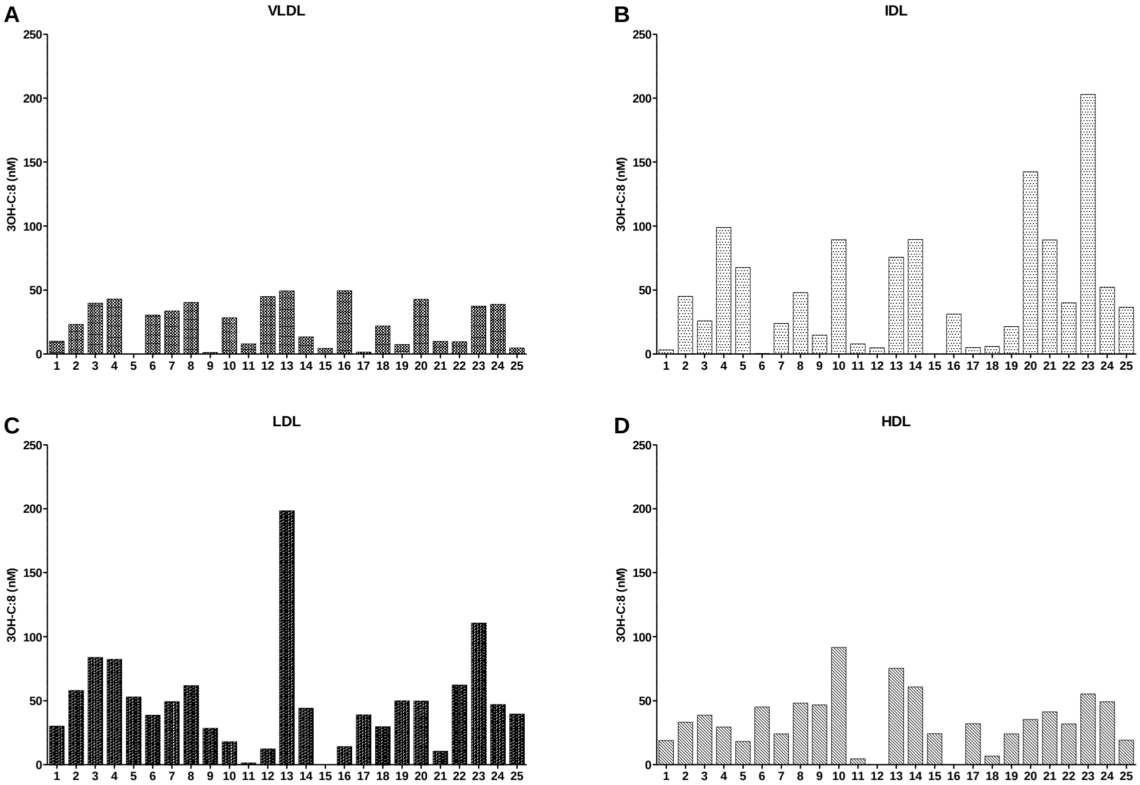

Supplemental figure S3: Individual concentrations (nM) of 3OH-C:8 fatty acid in VLDL (A), IDL (B), LDL (C) and HDL (D) fractions of all study subjects.

Supplemental figure S4

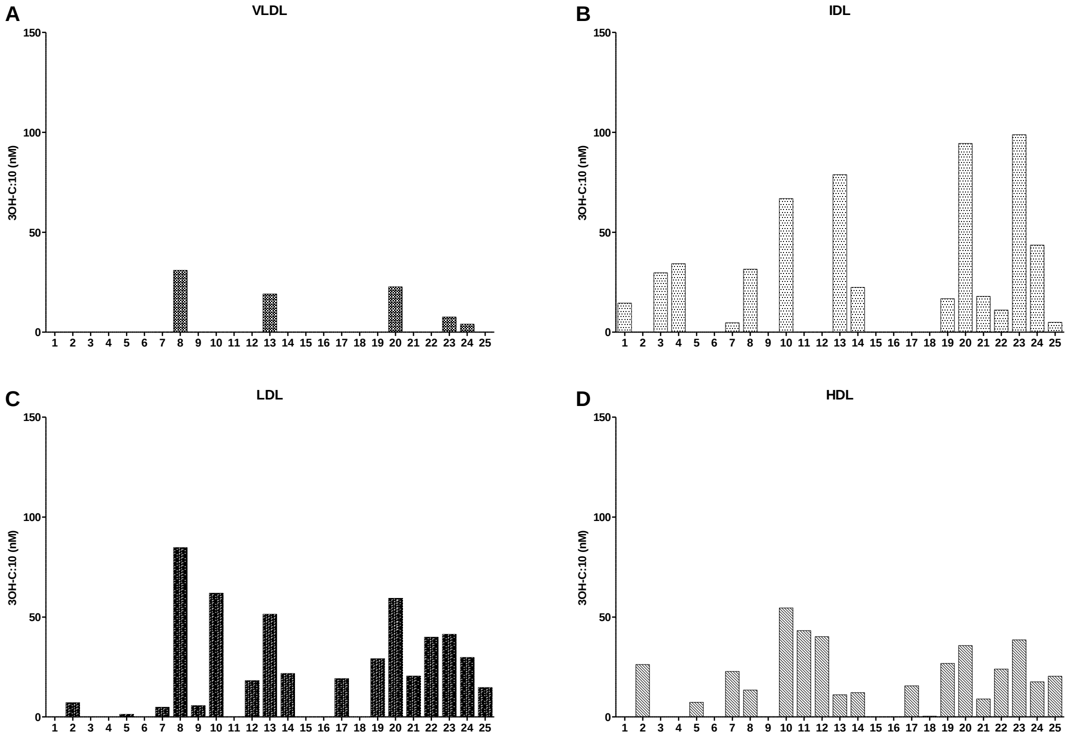

Supplemental figure S4: Individual concentrations (nM) of 3OH-C:10 fatty acid in VLDL (A), IDL (B), LDL (C) and HDL (D) fractions of all study subjects.

Supplemental figure S5

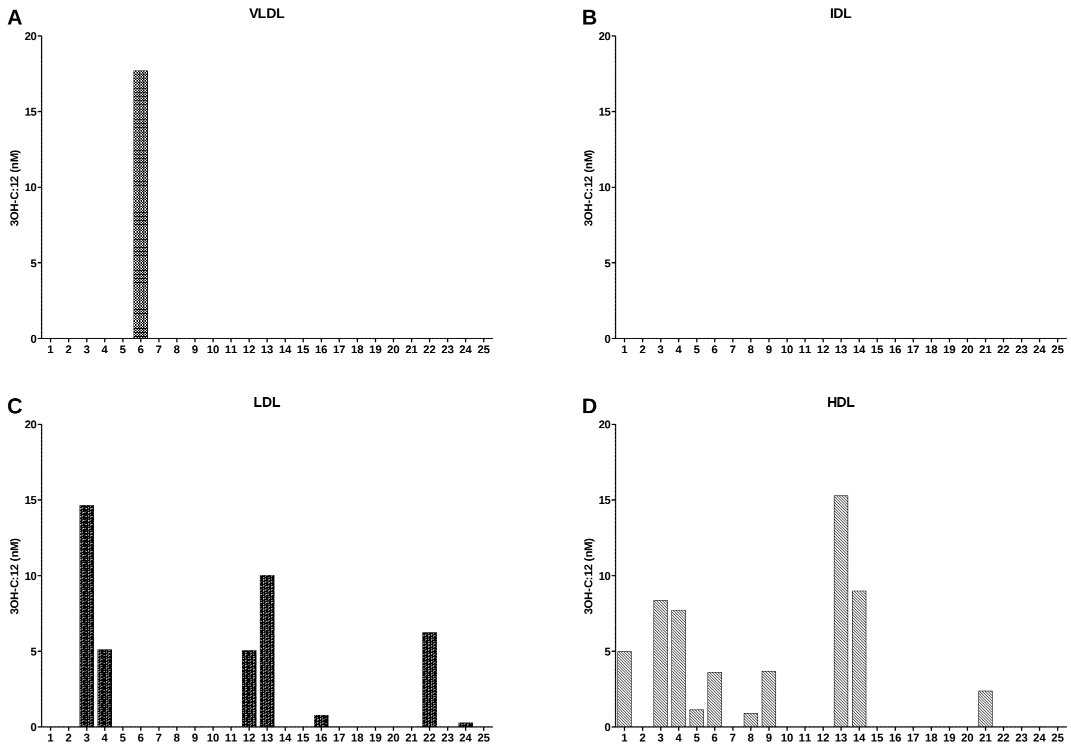

Supplemental figure S5: Individual concentrations (nM) of 3OH-C:12 fatty acid in VLDL (A), IDL (B), LDL (C) and HDL (D) fractions of all study subjects.

Supplemental figure S6

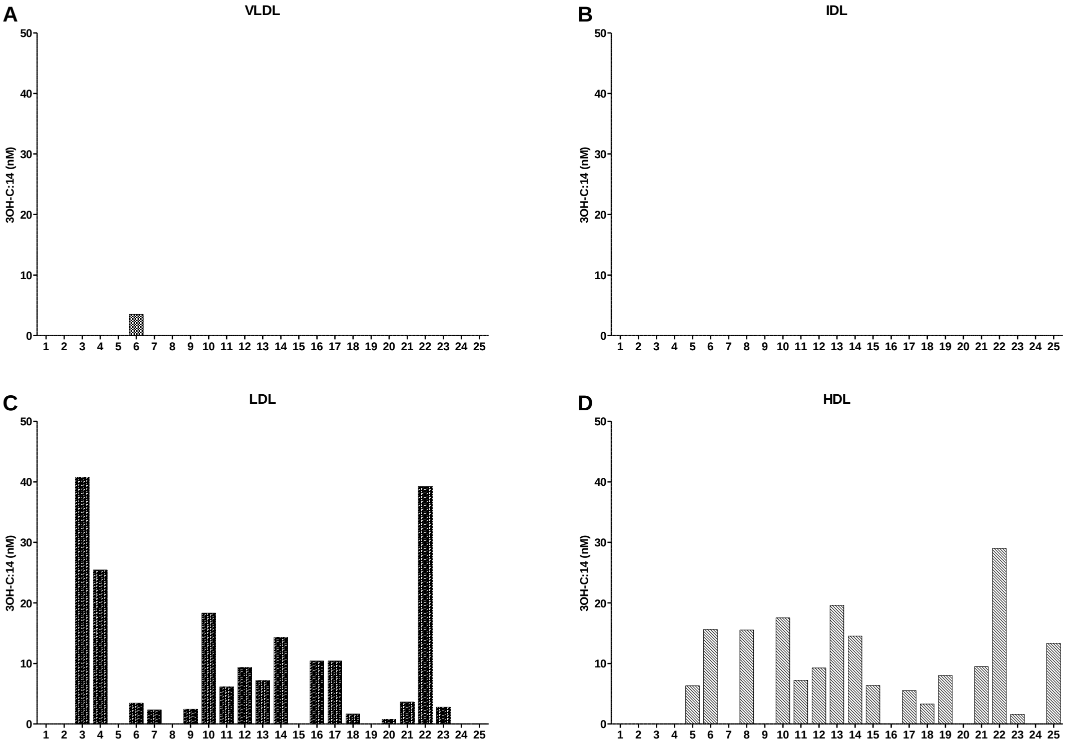

Supplemental figure S6: Individual concentrations (nM) of 3OH-C:14 fatty acid in VLDL (A), IDL (B), LDL (C) and HDL (D) fractions of all study subjects.

Supplemental figure S7

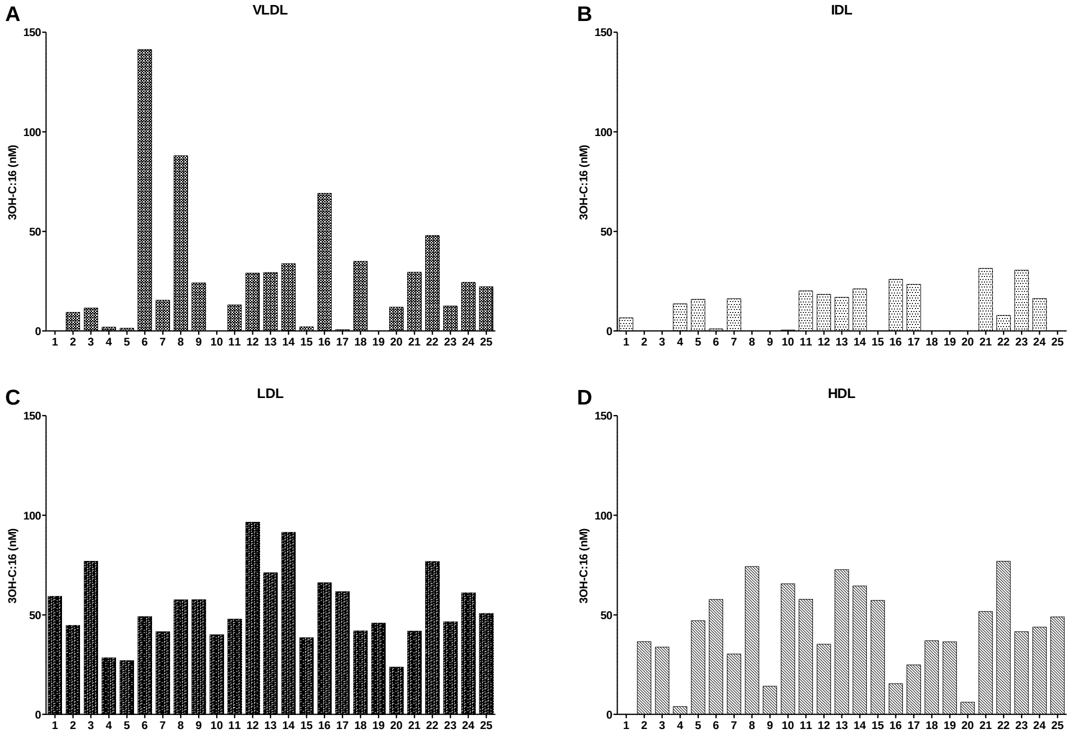

Supplemental figure S7: Individual concentrations (nM) of 3OH-C:16 fatty acid in VLDL (A), IDL (B), LDL (C) and HDL (D) fractions of all study subjects.

Supplemental figure S8

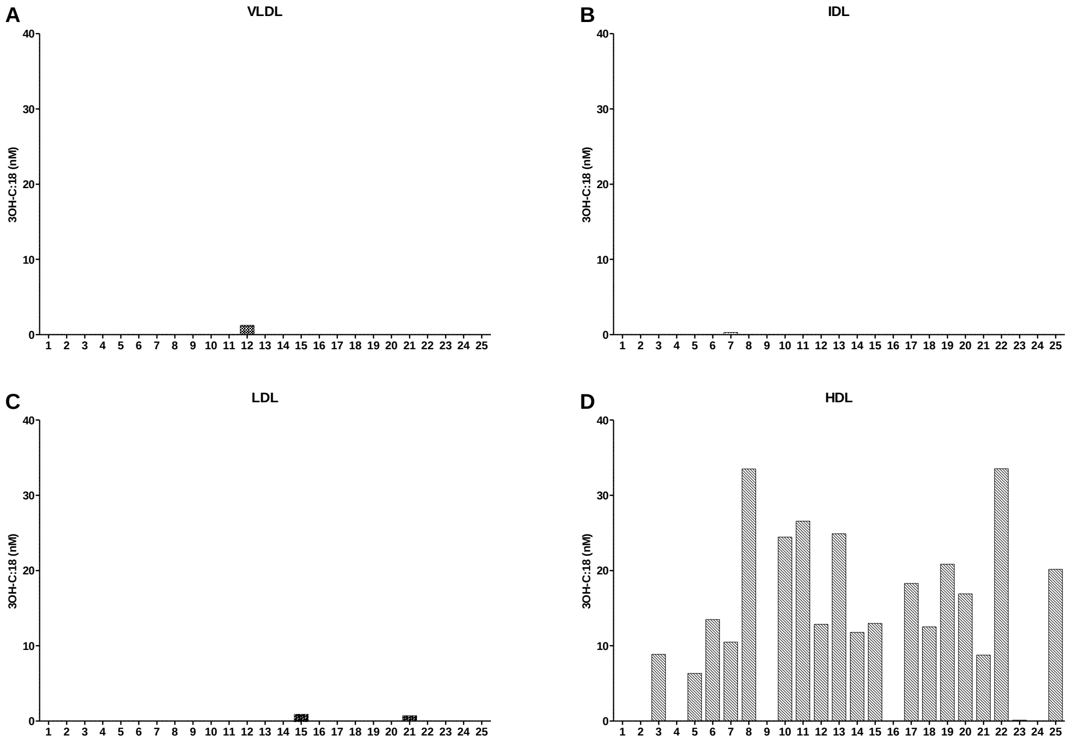

Supplemental figure S8: Individual concentrations (nM) of 3OH-C:18 fatty acid in VLDL (A), IDL (B), LDL (C) and HDL (D) fractions of all study subjects.
